# Gestational diabetes mellitus—right person, right treatment, right time?

**DOI:** 10.1186/s12916-017-0925-2

**Published:** 2017-08-28

**Authors:** Robert S. Lindsay, Sharon T. Mackin, Scott M. Nelson

**Affiliations:** 1 Institute of Cardiovascular & Medical Sciences, BHF Glasgow Cardiovascular Research Centre, University of Glasgow, University Place, Glasgow, G12 8TA UK; 2 School of Medicine, University of Glasgow, Glasgow Royal Infirmary, Glasgow, G31 2ER UK

**Keywords:** Gestational diabetes, Personalised medicine, Metformin, Glibenclamide

## Abstract

**Background:**

Personalised treatment that is uniquely tailored to an individual’s phenotype has become a key goal of clinical and pharmaceutical development across many, particularly chronic, diseases. For type 2 diabetes, the importance of the underlying clinical heterogeneity of the condition is emphasised and a range of treatments are now available, with personalised approaches being developed. While a close connection between risk factors for type 2 diabetes and gestational diabetes has long been acknowledged, stratification of screening, treatment and obstetric intervention remains in its infancy.

**Conclusions:**

Although there have been major advances in our understanding of glucose tolerance in pregnancy and of the benefits of treatment of gestational diabetes, we argue that far more vigorous approaches are needed to enable development of companion diagnostics, and to ensure the efficacious and safe use of novel therapeutic agents and strategies to improve outcomes in this common condition.

## Background

May 2018 will mark 10 years since the publication of the seminal Hyperglycemia and Adverse Pregnancy Outcomes Study (HAPO) [1] and 8 years since new criteria and pathway for diagnosis of gestational diabetes (GDM) were proposed by the International Association of Diabetes and Pregnancy Study Groups (IADPSG) [2]. Landmark studies of the treatment of 'mild' GDM had been published before this in 2005 [3] and 2009 [4]. The IADPSG criteria have been largely adopted by several national and international societies, notably by the World Health Organisation (WHO) [5] and International Federation of Gynecology and Obstetrics (FIGO) [6], but others, most influentially the American College of Obstetricians and Gynecologists (ACOG) [7] and the National Institute for Health and Care Excellence (NICE) [8] have not adopted the criteria. Although much has been achieved, it is perhaps disappointing that the goal of a more unified pattern of screening and diagnosis has not been realized. This is especially true given that there is now overwhelming evidence that there is a graded linear association between either fasting or post-load glucose concentrations and both fetal growth and important adverse perinatal outcomes [9].

The lack of an inflection point at which risk increases means that decisions regarding diagnostic thresholds may be reasonably disputed, but setting some threshold for clinical decision-making is unavoidable. Critically, however, those thresholds should be set on the basis of defined clinical risks and benefits, with clear-sighted agreement as to whether these apply to perinatal and longer-term outcomes. In turn, it would appear that the use of individual data and calculated risk would be the most effective way in which to apply such a threshold.

By whichever criterion we diagnose GDM, its impact and that of associated metabolic disorders, such as type 2 diabetes mellitus (T2DM), in pregnancy grows ever more concerning. Rates of T2DM and obesity in pregnancy continue to increase. As the HAPO trial made clear, maternal glucose is one risk factor that predicts a continuum of risk, but additional risk factors such as body mass index (BMI) can also have an independent effect [1, 10]. Here, we review some recent developments in the prevention, screening, diagnosis and treatment of GDM and argue that efforts to individualise these processes should be central to the next ten years of development in this area.

## Preventing gestational diabetes: right person, right treatment?

GDM is associated with a number of well-described risk factors. The past few years have been notable for a number of excellently conducted trials that have attempted to determine whether intervention with exercise, diet or lifestyle, or pharmaceutical intervention with metformin, might reduce the incidence of GDM in pregnancy. These are potentially attractive options, not least as the concept of lifestyle modification, for example smoking cessation, during pregnancy is well accepted. Exercise has a number of known benefits in pregnancy and is already recommended [11]. Unfortunately, it has been surprisingly difficult to find an effective intervention to reduce the risk of GDM.

In the two largest lifestyle intervention trials to date, LIMIT (*n* = 2152 overweight and obese women) and UPBEAT (*n* = 1555 obese women), exercise and dietary change did not reduce either GDM or rates of large for gestational age babies [12]. More generally, while diet or exercise or their combination may reduce gestational weight gain modestly [13], it is far from clear that this reduction translates into a reduction in GDM when applied to more general populations [14, 15]. This has been seen most recently in the DALI (vitamin D and lifestyle intervention) trial of diet and physical activity, during which a combination of these interventions was successful in limiting gestational weight gain but did not alter fasting glycaemia in women at risk of GDM [16]. Similarly, the two large intervention trials administering metformin in pregnancy in obese women (N = 449 and N = 450) have failed to show any reduction in the incidence of GDM [17, 18]. There are some trials suggesting a positive effect of lifestyle intervention on GDM prevention. In the ROLO trial (N = 800 women with previous large for gestational age babies), a low glycaemic index diet was associated with a reduction in maternal glucose intolerance [19]. In the RADIEL trial (n = 293 women with a history of GDM and/or obesity), incidence of GDM was reduced by 39% with lifestyle intervention [20]. Notably, for the negative trials, inclusion was primarily predicated on BMI, rather than on a more sophisticated stratification of risk. For ROLO, secondary analysis identified that those women who were more likely to respond to the intervention had a slightly lower BMI, greater insulin resistance in early pregnancy, and lower circulating leptin at baseline [21]. RADIEL included a relatively high proportion of women with GDM in a previous pregnancy (30–35% [20]), which raises the possibility that differences in the underlying patterns of risk factors might underpin the discordance in results despite the similarity in intervention. It is also possible that particular types of diet, most notably a Mediterranean diet, may be helpful but data are largely limited to observational series [22].

The concept that T2DM is a heterogeneous condition, with multiple phenotypes and pathological pathways attached to a common disease label, is now a very familiar one [23]. Despite the strong association between GDM and future risk of T2DM, there has been less consideration of the possibility that GDM may also reflect a relative umbrella term with multiple threads. That the heterogeneous pathophysiologies of these two conditions may also differ has not been widely considered. The concept that T2DM is preceded by increases in liver and beta cell fat content (triacylglycerol), and that this can be reversed by weight loss, has become influential recently [24]. By contrast, GDM does not appear to be associated with an increase in liver triacylglycerol [25]. Furthermore, in a small study of 14 women with GDM, calorie restriction over a 4-week period reduced liver triacylglycerol from what were apparently already normal levels, but this reduction was still associated with a reduced need for pharmacological therapy as compared to matched controls [25]. Taken together, these findings may suggest, first, that there are differences in pathophysiology, perhaps explaining why lifestyle and pharmacological interventions have had inconsistent effects in comparison to similar interventions to prevent T2DM. Second, the evidence suggests that we need to understand the heterogeneity of GDM risk in individuals in order to facilitate the targeting of preventative strategies.

## Screening and diagnosis: right person, right time?

There has been extensive debate on which screening and diagnostic criteria are most appropriate. That debate has been extensively aired elsewhere [26] and the risk characteristics of patients diagnosed under one or other system examined [27]. It can be noted that recommendations still vary as to whether all women or only women with risk factors (usually including a measure of obesity or overweight, previous macrosomia, ethnicity, and/or family history of diabetes or polycystic ovary syndrome [PCOS]) are screened biochemically [2, 5, 6]. Inevitably, any pre-selection that is based on any single risk factor, which has in itself only a modest association with GDM, will perform relatively poorly. So although stratification of screening to identify those at greatest risk may seem attractive, the efficacy of this approach will be limited and universal screening is more efficacious in populations with a high prevalence of any individual risk factor. An alternative approach is to try to develop more accurate multivariate models to identify those at risk, particularly as early pregnancy models would also enable early targeted intervention [28]. Although multiple biomarkers that have been associated with T2DM have been examined, circulating metabolic measures such as assessment of adiponectin levels appear to be most promising [29]. Evaluation of a novel early pregnancy screening algorithm and an intervention could be undertaken simultaneously [30].

An alternative approach is to shift the timing of the diagnostic test that confirms GDM. Traditionally, screening and diagnostic thresholds are applicable at 24 to 28 weeks. This makes some sense in terms of the dynamics of change of glucose in pregnancy, but limits the period during which intervention can occur. More subtle metabolic perturbations can be identified prior to the diagnosis of GDM or early in pregnancy in obese women, and what is more striking is that these changes have a biological consequence. The POP study showed that fetal growth is increased even at the time of diagnosis of GDM at 28 weeks, an increase that is not apparent at 20 weeks [31]. This adds weight to the logic of questioning the timing of current GDM diagnosis and suggests that an opportunity for intervention might be being missed. Even more strikingly, growth is already increased in the fetuses of obese women by 20 weeks gestation, suggesting that successful interventions might need to occur even earlier in this group if growth is to become normal [31].

As an additional issue, although the original IADPSG report suggested that the new criteria could also be used in screening in early pregnancy [2], this approach has been controversial. There is an undoubted clinical need to find women with undiagnosed diabetes in early pregnancy, especially in populations with high prevalence of undiagnosed T2DM. Nevertheless, the most clinically relevant and cost effective way of doing this is not clear at present. A separate aim is to identify women who might benefit from early intervention to prevent GDM. This could be achieved relatively easily as a research study. Testing both during early pregnancy and again at 24–28 weeks, with the identification of appropriate first-trimester values that are sufficiently sensitive and specific to allow them to replace agreed IADPSG or alternative thresholds at 24–28 weeks, would enable the field to move forward. Alternative novel biomarkers that could replace the dynamic oral glucose tolerance test could then be validated on the same large cohort and associated biobank.

## Treating gestational diabetes: right treatment?

The cornerstone of treatment of GDM remains dietary intervention. Other positive lifestyle changes including increased exercise are also encouraged. Lifestyle modification is a critical component of GDM treatment, and it should be remembered that the successful treatment trials of 'mild' GDM involved treatment protocols that relied on dietary therapy alone in over 80% of cases [3, 4]. The nutrition recommendations of the American Diabetes Association (ADA) were followed [4]. After the year 2000, treatment underwent a major change in many countries with the trials of glyburide (also known as glibenclamide) [32] and metformin [33], allowing use of these agents usually prior to insulin therapy. Treatment selection is relatively unsophisticated: most patients will begin with diet, then proceed to an oral hypoglycaemic and finally progress to insulin treatment. That insulin is frequently required even with use of oral hypoglycaemics suggests either that the glycaemic challenge of pregnancy is too great for the oral hypoglycaemics used at present or that there is a potential mismatching of the optimal oral therapy with the patient profile [32, 33]. The unique safety concerns associated with potential transplacental transfer and effects on the fetus have limited the evaluation of efficacious pharmacological treatments such as dipeptidyl peptidase-4 inhibitors, sodium–glucose cotransporter 2 inhibitors or GLP-1 mimetics, which are routinely used for T2DM. This limitation is likely to continue. As for the use of other non-licensed medications during pregnancy, unplanned exposure in early pregnancy will allow only some, imperfect assessment of safety. The potential weight loss associated with some of these agents should also limit use in pregnancy and mandates only careful trials for their evaluation. It would seem likely, however, that pharmaceutical companies will be reluctant to take on the expense and possible risk of such trials.

Can we select which women are best suited for one or other of the currently available oral therapies? The evidence base is small and so outcomes in subgroups have not been reported extensively. When considering glucose-lowering effects, the existing randomized control trial and observational series suggest that the failure of glibenclamide is more likely when initial fasting glucose is high (above 6.4 mmol/l) [34]. These observations make sense as women with higher glucose levels are likely to have more severe disease. Similarly, in observations of metformin use, women who required supplemental insulin had higher BMI in early pregnancy than those maintained on metformin (33.6 ± 8.6 kg/m^2^ vs 31.1 ± 7.8 kg/m^2^ ); likewise, baseline glucose levels were higher in those requiring supplemental insulin (6.1 ± 1.1 mmol/l) than in those not requiring supplemental insulin (5.3 ± 0.8 mmol/l) [33]. Metformin does have an advantage in being associated with less weight gain than either insulin or glibenclamide, and it may be more attractive in general for this reason [33]. In the future, pharmacogenetics might help with this selection, but large studies will be needed and evidence of robust effects for metformin is currently lacking, even in type 2 diabetes [35]. More broadly, several studies have attempted to predict which women might need insulin therapy for GDM. A range of factors—maternal age, family history of diabetes, obesity, prior GDM, early diagnosis of GDM, higher fasting venous blood glucose level and HbA1c—have been shown to be predictive and may be useful to guide intensity of follow-up [36].

The relative benefits of the various agents used to reduce complications, such as pre-eclampsia, are less clear. Pre-eclampsia risk in GDM is 1.5-fold greater than that of the background population [37] and treatment of 'mild' GDM reduces risk of preeclampsia by 30–32% [3, 4], a greater benefit than that provided by other preventative strategies such as low-dose aspirin (which provides a more modest 10% risk reduction in high-risk pregnancy) [38]. Meta-analyses have suggested additional benefit with metformin therapy compared with insulin or glibenclamide treatments in preventing pregnancy-induced hypertension [39, 40], but this is only a non-significant trend towards lower pre-eclampsia rates seen in studies that are underpowered in their ability to test this association. Examination of whether such benefits can be extended to other patient groups remains at an early stage [17, 18]. In general, the mechanisms of how reduced glucose levels alter preeclampsia risk, whether this mechanism differs between agents and, in turn, whether this should influence therapeutic choices and patient selection remain understudied issues.

A further contentious area has been the risk of neonatal hypoglycemia with glibenclamide use. While initial studies suggested that glibenclamide does not cross the placenta at significant levels [41], studies have now shown that it does cross the placenta but is then actively effluxed out of the fetal unit by a specific transporter (placental breast cancer resistance protein) [42]. Polymorphism in this protein can lead to variable levels of glibenclamide in the fetus [42]. This, along with the observation that neonatal outcomes may be inferior with glibenclamide, particularly for neonatal hypoglycemia [34, 39], has led bodies such as the ADA to recommend insulin as the first-line agent after diet [43]. The ADA also notes the lack of long-term safety data for either metformin or glibenclamide in that recommendation [43]. Both agents cross the placenta and so there is potential for long-term programming effects, either directly by the drug or, in the case of glibenclamide, by induction of fetal hyperinsulinaemia. Metformin is known to increase adenosine monophosphate-activated protein kinase (AMPK) and increases in AMPK may be important in utero-; for example, they are important in diabetic embryopathy [44]. Importantly, animal studies have not suggested an increase in embryopathy with early metformin exposure in vivo [44], and meta-analyses of human studies based on metformin exposure in women with PCOS also do not suggest any increase in congenital anomalies [45]. Nevertheless, there could be a question as to whether metformin exposure will have longer-term effects.

Randomized evidence arising from exposure during pregnancy extends only to 2 years, but is largely reassuring. In data from the MIG study (MIG TOFU), children exposed to metformin in utero had normal total fat mass and percentage body fat as assessed by bioimpedance, although these children did have slightly larger mid-upper arm circumferences and subscapular and biceps skinfolds [46]. There were no differences in blood pressure [47]. Follow-up of a Finnish randomized controlled trial (RCT) found that children who were exposed to metformin in pregnancy were significantly heavier at the age of 12 months, and both taller and heavier (12.0 vs 11.3 kg) at 18 months. The mean ponderal index did not differ significantly. Motor, social and linguistic development evaluated at the age of 18 months did not differ between the groups [48]. A recent review noted the limitations of the size of the study and the length of follow-up in the available studies [40]. Different clinicians are likely to interpret this evidence base differently when recommending glibenclamide, metformin or insulin to their patients. Overall, it is important to note that, in recent years, we have been fortunate to have detailed RCTs examining different agents in pregnancy. Given the heterogeneity of T2DM and GDM, it would be useful to have a wider range of therapies. At present, the pharmacological resources available appear to be becoming more rather than less limited, as glibenclamide use declines.

Finally, there is limited information comparing different treatment targets, independent of which treatment is used to lower glucose, particularly for type 1 and type 2 diabetes. In GDM, the interventional trials [3, 4] followed set algorithms for escalation of treatment (for example, treatment escalated when fasting glucose exceeded 5.5 mmol/l or when glucose values two hours after meals exceeded 7.0 mmol/l) but few studies have compared different targets. The study of de Veciana et al. [49] supported the importance of using post-prandial rather than pre-prandial targets for insulin adjustment in GDM using a randomised design leading to the wider adoption of post-prandial monitoring.

## Obstetric decision making: right monitoring and timing of delivery?

With the potential tsunami of GDM, antenatal care pathways that classically stratify GDM into a high-risk care pathway need to be reconsidered. Routine antenatal visits primarily focus on detecting hypertensive disorders of pregnancy and abnormalities of fetal growth, which are substantially less prevalent than GDM. An alternative model that enables GDM-affected women who only receive lifestyle modification or oral agents to continue in their respective non-specialist services can be attainable with appropriate education of healthcare providers. The cost of instruction, of the consumables used for frequent self-monitored blood glucose (SMBG) assessments and of more intensive antenatal care are the greatest costs of antenatal GDM provision. These costs potentially nullify the cost-effectiveness of GDM screening and treatment, suggesting that alternative more sophisticated approaches are required. The purpose of the SMBG is to identify those that will benefit from intensification of therapy, but it also facilitates learning and reinforcement of the importance of an optimal diet. Validated treatment escalation algorithms (indicating glucose levels at which treatment is increased) [3, 4] and remote management of SMBG monitors (which can be used with the smart devices that are routinely carried by patients of this age-group) may facilitate a reduction in patient inconvenience and the need for widespread attendance at specialist services.

The second greatest contribution to the antenatal costs of GDM management is the inclusion of additional ultrasounds for fetal growth. Long-standing opinions regarding the lack of merit of routine ultrasound for fetal growth from 24 weeks onwards are now being challenged [50]. Data from blinded ultrasound measurements, performed at 28 weeks and 36 weeks in nulliparous women, show that the identification of fetuses with an estimated fetal weight of less than the 10^th^ percentile and an abdominal circumference growth velocity in the lowest decile can identify those small for gestational age fetuses at increased risk of neonatal morbidity [51]. Adoption of these two scans into current routine care would, first, enable identification of GDM-affected fetuses with abnormal growth at the time of diagnosis and ensure early intensification of treatment. Second, the identification at 36 weeks of fetuses that have impaired growth due to placental dysfunction later in pregnancy and would identify those that would benefit from expedited delivery, while balancing against widespread iatrogenic premature delivery. Conversely, for those fetuses with an anticipated estimated fetal weight above the 95^th^ centile, induction of labor at 37 to 38 + 6 weeks is associated with a reduced risk of shoulder dystocia and associated morbidity as compared to expectant management [52]. The value of having an additional third scan at 32 weeks may be limited in this context because SMBG monitoring rather than ultrasound indices would primarily indicate the need for treatment escalation. Similarly, detection of abnormal growth to the extent that would precipitate premature delivery is unlikely in the absence of alteration of SMBG monitoring or other clinical signs. In many healthcare systems, routine ultrasound at all three time points is already provided, in which case there is essentially no increase in the ultrasonographic cost of GDM.

## Conclusions

We all wish for personalised care that accounts for our underlying genotype, phenotype and environment. For GDM, we are beginning to understand how and when to identify and monitor those at greatest risk and to intervene appropriately. We should now aspire to remove the need for treatment escalation while ensuring that every patient gets the right treatment from the moment of diagnosis and attains an optimal pregnancy outcome. The need for such a personalised approach is particularly pressing in GDM: in resource-limited health care systems it is mandatory that we concentrate testing and resources where we can demonstrate that the most benefit will occur. An individualised approach to screening, treatment and monitoring can be the only way ahead.

## Abbreviations

ADA: American Diabetes Association
AMPK: Adenosine monophosphate-activated protein kinase
BMI: Body mass index
FIGO: International Federation of Gynecology and Obstetrics
GDM: Gestational diabetes mellitus
HAPO: Hyperglycemia and Adverse Pregnancy Outcomes Study
IADPSG: International Association of Diabetes and Pregnancy Study Groups
PCOS: Polycystic ovary syndrome
RCT: Randomized controlled trial
SMBG: Self-monitored blood glucose
T2DM: Type 2 diabetes mellitus
